# Skeletal System Biology and Smoke Damage: From Basic Science to Medical Clinic

**DOI:** 10.3390/ijms22126629

**Published:** 2021-06-21

**Authors:** Umberto Tarantino, Ida Cariati, Chiara Greggi, Elena Gasbarra, Alberto Belluati, Luigi Ciolli, Giulio Maccauro, Alberto Momoli, Simone Ripanti, Francesco Falez, Maria Luisa Brandi

**Affiliations:** 1 Department of Clinical Sciences and Translational Medicine, “Tor Vergata” University of Rome, Via Montpellier 1, 00133 Rome, Italy; umberto.tarantino@uniroma2.it (U.T.); ida.cariati@uniroma2.it (I.C.); chiara.greggi@gmail.com (C.G.); gasbarra@med.uniroma2.it (E.G.); 2 Department of Orthopaedics and Traumatology, “Policlinico Tor Vergata” Foundation, Viale Oxford 81, 00133 Rome, Italy; 3 Medical-Surgical Biotechnologies and Translational Medicine, “Tor Vergata” University of Rome, Via Montpellier 1, 00133 Rome, Italy; 4 Orthopaedic and Traumatology Department, Hospital Santa Maria delle Croci–AUSL Romagna, Viale Randi 5, 48121 Ravenna, Italy; dr.belluati@libero.it; 5 Orthopaedic and Traumatology Department, S. Spirito Hospital, Lungotevere in Sassia 1, 00193 Rome, Italy; lui.ciolli@gmail.com (L.C.); francesco.falez@francescofalez.it (F.F.); 6 Department of Orthopaedics and Traumatology, Fondazione Policlinico Universitario A. Gemelli IRCCS, Università Cattolica del Sacro Cuore, Largo Agostino Gemelli 8, 00168 Rome, Italy; giulio.maccauro@unicatt.it; 7 Orthopedic and Traumatology Department, San Bortolo Hospital-AULSS 8 Berica, Viale Rodolfi 37, 36100 Vicenza, Italy; alberto.momoli@gmail.com; 8 Department of Orthopaedics and Traumatology, San Giovanni-Addolorata Hospital, Via dell’Amba Aradam 8, 00184 Rome, Italy; simone.ripanti@gmail.com; 9 FIRMO Foundation, 50141 Florence, Italy

**Keywords:** smoking, bone metabolism, fracture healing, surgical complications, smoking cessation

## Abstract

Cigarette smoking has a negative impact on the skeletal system, as it reduces bone mass and increases fracture risk through its direct or indirect effects on bone remodeling. Recent evidence demonstrates that smoking causes an imbalance in bone turnover, making bone vulnerable to osteoporosis and fragility fractures. Moreover, cigarette smoking is known to have deleterious effects on fracture healing, as a positive correlation between the daily number of cigarettes smoked and years of exposure has been shown, even though the underlying mechanisms are not fully understood. It is also well known that smoking causes several medical/surgical complications responsible for longer hospital stays and a consequent increase in the consumption of resources. Smoking cessation is, therefore, highly advisable to prevent the onset of bone metabolic disease. However, even with cessation, some of the consequences appear to continue for decades afterwards. Based on this evidence, the aim of our review was to evaluate the impact of smoking on the skeletal system, especially on bone fractures, and to identify the pathophysiological mechanisms responsible for the impairment of fracture healing. Since smoking is a major public health concern, understanding the association between cigarette smoking and the occurrence of bone disease is necessary in order to identify potential new targets for intervention.

## 1. Introduction

Tobacco smoking is known to have negative consequences on most systems of the human body and it is considered one of the main risk factors for the occurrence of non-communicable diseases, causing around 6 million deaths each year [1,2,3,4]. In addition, despite the annual decline in smoking prevalence, the number of people smoking has increased due to population growth [5].

In recent years, extensive studies have been conducted on the association between smoking and musculoskeletal disorders, confirming the existence of a causal relationship between tobacco smoking and rheumatoid arthritis, periodontitis, osteoporosis, and fragility fractures [6]. This association is related to the effects of smoking on imbalances in bone turnover, with a consequent increase in bone fragility [7,8]. Moreover, numerous studies have shown how long-term smoking is associated with a decline in muscle functionality and sarcopenia [9,10].

Based on this evidence, in this review we have evaluated the most recently published literature and summarized the scientific evidence regarding the effects of smoking on the skeleton, with the aim of analyzing the accumulated knowledge on the underlying molecular and cellular mechanisms.

## 2. Smoking and Bone Damage

Cigarette smoking is known to have deleterious effects on bone integrity, as a positive correlation between the daily number of cigarettes smoked and years of exposure has been shown [11,12]. To date, it has not yet been identified which of the various toxic compounds present in cigarette smoking are responsible for the negative effects of smoking on bone metabolism. However, several researchers attribute such undesirable effects to nicotine, one of the main components of the particulate phase of tobacco smoke [13].

Cigarette smoking is also considered one of the main social risk factors for developing metabolic bone diseases (such as osteoporosis, Paget’s disease, osteomalacia, and diabetic osteopathy), which compromise bone remodeling for a variety of reasons, including altered bone formation by osteoblasts, increased bone degradation by osteoclasts, or a combination of both [12,14,15]. In particular, the first suggestion of an association between tobacco smoking and osteoporosis dates to a study conducted in 1976, which showed that the onset of advanced idiopathic osteoporosis occurred before age 65 with a significantly higher percentage in smokers versus non-smokers [16]. Subsequently, the effect of smoking on bone mineral density (BMD) has been confirmed by several epidemiological studies. In 2002 Gerdhem and Obrant evaluated BMD using dual energy X-ray absorbimetry (DEXA) in a cohort of 19,500 subjects, with 14,000 showing significantly lower BMD values in the femoral neck and to a lesser extent in the lumbar spine [11]. More recently, a five-year longitudinal study was carried out in a population of 833 young men, aged between 18 and 20 years, evaluating bone density and geometry using high-resolution peripheral quantitative computed tomography [17]. Rudäng et al. observed that men who start smoking at a young age reach a lower BMD at several sites, as well as a lower trabecular density and smaller cortical cross-sectional area, than their nonsmoking peers, indicating that starting smoking impairs bone mass development in young adulthood [17].

It is generally accepted that BMD loss is related to hormonal imbalance, aging, environmental factors, lifestyle, and genetic predisposition [18]. Bączyk et al. conducted a study to determine which sociodemographic and clinical parameters represent risk factors for reduced BMD in 41 pairs of female twins who are discordant in therms of their smoking habit [19]. The study showed that women who had smoked one pack of cigarettes every day during adulthood had an average bone density deficit of 5–10% at the time of menopause, which is sufficient to increase the risk of fracture [19]. Similarly, an epidemiological study conducted by Noale et al. in 2012 on post-menopausal women showed that smokers lose significantly more cortical bone than non-smokers [20].

## 3. Pathophysiological Mechanisms of Smoking Effects on Bone Tissue

The pathophysiological mechanisms by which smoking affects bone health are still unclear, as the direct and indirect effects of smoking on bone remodeling are numerous (Table 1).

Among the indirect mechanisms through which smoking exerts a negative effect on bone mass, there is the alteration of body weight [21]. In fact, several studies in the literature have shown that there is an inverse relationship between smoking and body weight [22,23,24]. Effects of smoking on body weight are due to nicotine, which suppresses appetite and promotes the increase in serum concentrations of dopamine and serotonin, with the inhibition of food ingestion [25]. In addition, nicotine seems to have a direct effect on fat metabolism, leading to an increase in lipid oxidation and, consequently, a decrease in body mass index (BMI) [26]. According to Wong and colleagues, the alteration of body weight caused by tobacco consumption produces a reduced BMD and an increased risk of fractures through three likely mechanisms: (i) weight loss causes a decrease in the mechanical load on the bone, leading to a reduction in the osteogenic stimulus; (ii) in smokers the adipose tissue reduction is followed by a reduced conversion of androgens into estrogens, which is known to have positive effects on bone mass; and finally (iii) in smokers the decreased levels of leptin produce a reduced bone mass [27]. An association between cigarette smoking and sarcopenia has also been reported as an indirect effect. Compounds present in cigarette smoke have been shown to degrade proteins in muscle tissue [28,29].

The parathyroid hormone (PTH)–vitamin D axis, which plays a fundamental role in determining BMD and calcium homeostasis, is also affected by smoking [30]. PTH regulates serum calcium levels through bone and kidney reabsorption, whereas vitamin D regulates the absorption of calcium and phosphate at the intestinal level [31]. Epidemiological, in vitro, and in vivo studies have shown that smokers compared to non-smokers, show low vitamin D and PTH serum levels, exhibiting a suppressive effect of tobacco on the production of PTH, cholecalciferol, and calcitriol [32]. Moreover, Kassi and colleagues have shown that this association is independent of age or gender, demonstrating that the probability of having a vitamin D_3_ deficiency was increased from 58% to 63% in smokers compared to non-smokers independently of sex and age [33]. Among the mechanisms that could determine the observed decrease in vitamin D levels, skin aging can be considered, as smoking plays a predominant role in the skin aging process [32]. Lahmann and colleagues showed that the underlying mechanism for skin senescence is the activation of metalloproteinases (MMPs) in smokers compared to non-smokers [34].

It is known that gonadal hormones influence bone remodeling, with testosterone directly increasing osteoblast proliferation (through the androgen receptors present on their surface) and with estrogen suppressing osteoclasts activity. The effects of smoking on testosterone are controversial. According to many studies, smoking does not seem to have an effect on total testosterone levels, which remain unchanged between smokers and non-smokers; whereas according to others, there is a direct correlation between serum testosterone levels and nicotine levels [35]. In women, smoking has a clear antiestrogenic effect through different actions [36]. First, there is a reduction in the aromatization of androgens to estrogens, and this is evident after menopause. Second, researchers have documented a negative effect of smoking on reproductive organs [37], with the direct inhibition of aromatase activity in granulosa cells by some alkaloid derivatives of tobacco [38]. Another proposed effect of smoking is the 2-hydroxylation of estradiol, resulting in 2-hydroxyestrogens, which have minimal estrogenic activity and which are immediately removed from circulation [39].

Finally, among the most significant effects of smoking is the increased release of cortisol into the circulation due to an upstream increase in the levels of circulating adrenocorticotrophin (ACTH). As increased high levels of circulating cortisol correlate with an increased risk of osteoporosis, this could represent a causal indirect effect of smoking contributing to the reduction of BMD [40,41].

Furthermore, according to Stalke and colleagues, nicotine may exert an anti-diuretic effect by modulating plasma vasopressin (AVP) levels. Specifically, they observed a dose-dependent effect of nicotine on AVP, but also on ACTH and cortisol levels. At very low doses, no effect was observed; whereas at both intermediate and high doses, a significant increase in the plasma levels of all three hormones was observed [42]. In addition, tobacco consumption has also been shown to exert effects on adrenal androgen secretion; in fact, high levels of androstenedione and dehydropiandrosterone sulfate (DHEAS) are usually found in smokers [40].

Smoking exerts a direct effect on bone tissue via nicotinic acetylcholine receptors localized on the surface of osteoblasts. Based on the findings of Kim and colleagues, the effect is dose-dependent—at low concentrations, nicotine appears to have a stimulating effect on osteoblast proliferation, whereas at higher concentrations, the effect is inhibitory [43]. Cigarette smoke also exerts a direct effect on bone by altering bone-related factors involved in the regulation of osteoclastogenesis, such as the receptor activator of nuclear factor kappa-B ligand (RANKL)/receptor activator of nuclear factor kappa-B (RANK)/osteoprotegerin (OPG) system [44,45,46]. RANKL, released by osteoblasts, binds its receptor located on the surface of osteoclast precursor cells, promoting their differentiation and triggering bone resorption, whereas OPG, also released by osteoblasts, interacts with RANK, preventing the binding with its ligand and inhibiting the activity and proliferation of osteoclasts [47]. Several studies conducted on both animal and human models have shown that the RANKL/OPG ratio is higher in smokers than in non-smokers [21]. These results are in agreement with a study by Jorde and colleagues, who analyzed circulating bone turnover markers in subjects belonging to a population in northern Norway, showing that serum levels of dickkopf-1 (DKK1), an inhibitor of bone resorption through the WNT pathway, were increased in smokers, whereas the serum levels of procollagen type 1 amino-terminal propeptide (P1NP), a bone formation marker, were decreased [30].

## 4. Medical and Surgical Complications of Fragility Fractures in Smokers

Several researchers agree that smokers are at greater risk of infection [48,49]. In fact, the inhalation of the mixture of components present in tobacco smoke has negative effects on the immune system, both locally and peripherally [50]. Smoking is known to promote the infection of surgical wounds; this complication appears to be related to nicotine and carbon monoxide (CO), which are the main substances predisposing patients to wound infection [50]. Other components of cigarettes appear to reduce the deformability of erythrocytes, which is associated with endothelial lesions, thus blocking repair processes and promoting hypoxia and bacterial growth at the surgical site [51]. In this regard, it is now generally accepted that hypoxia and modifications in cellular metabolic activity are the main physiological changes occurring at the fracture site [52], mainly caused by nicotine and CO. Nicotine is known to be a powerful vasoconstrictor that causes a reduction in peripheral blood flow by increasing the production of thromboxane A2 and catecholamines and reducing the secretion of prostaglandin I2. On the other hand, CO is known to limit oxygen flow to tissues by inhibiting binding sites for hemoglobin (Hb). It has been hypothesized that a significant contribution to the deleterious effects of smoking is made through changes in blood supply that induce hypoxia, implying that the effect may be greater in severe fracture subtypes where oxygen delivery to the fracture site is more widely compromised [52].

Although it is not yet known with absolute certainty that the delay in the bone healing (union) after a fracture or surgery is directly related to smoking [53,54], there are several observational and experimental studies, both on human and animal models, aimed at demonstrating the existence of a strong association between the two phenomena [50]. According to most clinical reports, the effect of smoking is more evident in open fractures. For example, it was observed that patients with open and high-grade open fractures needed much more time to heal if they were smokers (32.3 weeks for smokers versus 27.8 weeks for non-smokers) [55]. On the other hand, other researchers argue that smoking is a risk factor for nonunion regardless of the open or closed nature of the fracture. In this regard, Hernigou and Schuind showed that smokers have a higher risk of developing a nonunion after a diaphyseal fracture of the humerus, femur, or tibia, in both open and closed forms [54].

## 5. Smoking and Fracture Healing

Cigarette smoking is known to increase the fracture risk and the burden on the healthcare system [56,57], delaying the fracture healing process, increasing the frequency of complications (e.g., infections and nonunion fractures), and prolonging hospital stays [11,12].

**Figure 1:**
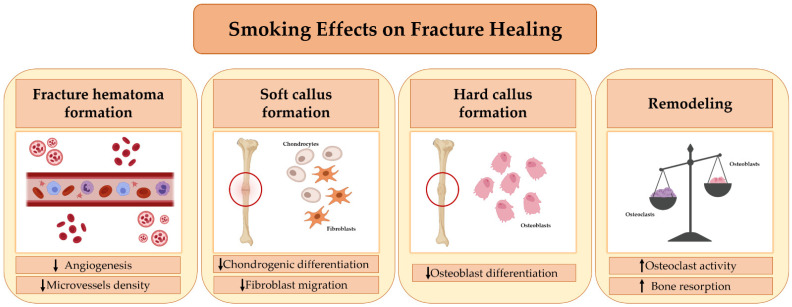
Smoking’s effects on Fracture Healing. Cigarette smoking affects every stage of fracture healing. First, it alters the formation of the fracture hematoma, impairing the process of angiogenesis. Second, it adversely affects the migration, proliferation, and differentiation of mesenchymal stem cells (MSCs) into chondrocytes, fibroblasts, and osteoblasts, impairing soft callus formation and the subsequent transition into a hard callus. Finally, smoking causes an imbalance between the activity of osteoblasts and osteoclasts, promoting the process of bone resorption and leading to a delay in fracture healing.

Although in vitro and in vivo studies have been conducted over time on the effects of cigarette smoke inhalation on fracture healing, the underlying mechanisms have not yet been fully understood. According to some evidence, cigarette smoke can impair every step in the fracture healing process (Figure 1). In this regard, Chang et al. conducted a study to evaluate the effects of cigarette smoke on the formation of new blood vessels during the early stage of fracture healing [58]. The authors observed that cigarette smoke inhalation results in decreased expression of angiogenic markers, such as vascular endothelial growth factor (VEGF) and von Willebrand factor (vWF), during the early phase of bone healing, with a consequent decrease in the density of newly formed microvessels. Their findings suggest that smoking may impair healing by altering fracture hematoma formation [58].

Some researchers have suggested that cigarette smoking inhibits fibroblast migration, which is essential for callus formation and, more generally, for an efficient healing process [59]. In fact, it is well known that fibroblasts are important both for the deposition and remodeling of extracellular matrix and for the recruitment of growth factors and cytokines in the fracture site, where they trigger healing processes such as the formation of new vessels [60]. Aspera-Werz reported that cigarette smoke contains compounds that may affect osteogenic and chondrogenic differentiation [61]. Indeed, it has been observed that a decrease in serum levels of transforming growth factor-beta (TGF-β) occurs in smokers, impairing the recruitment, proliferation, and differentiation of mesenchymal stem cells (MSCs) in fibroblasts, osteoblasts, and chondrocytes. This effect, in turn, negatively affects the soft callus and hard callus formation phase [61]. Finally, cigarette smoking has been shown to alter bone turnover, causing an imbalance in the relationship between RANKL and OPG in favor of osteoclastogenesis and bone resorption [21]. This evidence suggests that cigarette smoking can also compromise the final phase of fracture healing, namely, bone remodeling [21]. Moreover, it has been observed that at bone healing sites, cigarette smoke inhalation modulates the gene expression of *alkaline phosphatase* (*ALP*), *bone morphogenetic protein-2* (*BMP-2*), *RANKL*, and *OPG* [62], signaling factors that are essential for the formation of new bone tissue.

Other hypotheses have been proposed to explain how smoking affects the fracture healing process, including low levels of antioxidants and vitamins and high levels of reactive oxygen intermediates in the circulation, as well as the attenuating effect of nicotine on endothelial nitric oxide synthase [63]. It has also been shown that nicotine decreases prostacycline production, a known vasodilator that is important in the fracture healing process [64].

Finally, it has also been reported that nicotine can at least partially contribute to delayed fracture healing by inhibiting tumor necrosis factor-alpha (TNF-α) secretion through activation of the cholinergic anti-inflammatory pathway. In particular, the nicotine released from smoking may act through the α7 nicotinic acetylcholine receptor (α7 nAChR) and activate the anti-inflammatory pathways, thus causing activation of the JaK2-STAT3 signaling pathway and inhibition of the NF-κB signaling pathway. As a result, the production of TNF-α is significantly reduced, which can lead to a prolonged soft callus stage and a decrease in the migration of MSCs in the bone healing process [65]. Based on this evidence, more studies are needed to understand the clinical treatment of nonunion fractures and to develop new therapies to accelerate bone healing in smokers.

## 6. Effects of Smoking Cessation on Bone Health

To date, there is little scientific evidence about the effectiveness of smoking cessation on bone health. Cornuz et al. examined the effects of cigarette smoking and smoking cessation on the risk of hip fracture in 116,229 women, aged between 34 and 59, who were followed up for 12 years [66]. The results of this study confirmed that smokers are at an increased risk of hip fracture, and this is proportional to cigarette consumption. Furthermore, it was observed that the risk decreased among former smokers, but without any apparent benefit until ten years after cessation. On the other hand, Gerdhem and Obrant speculated that smoking has a reversible effect on BMD, since they found an improvement in the BMD of former smokers in less than ten years [67]. Other studies confirmed the positive effects of smoking cessation on bone health, with an improvement in gonadal hormones, bone formation level, and resorption markers in six weeks and an improvement in BMD after one year of cessation/reduction in post-menopausal women [68,69].

In recent years, researchers have studied the impact of smoking cessation on fracture healing after the treatment of acute fractures. To date, there is no scientific evidence to show that quitting smoking after a fracture can improve healing; on the contrary, it has been observed that some of the consequences of smoking seem to continue for decades after cessation. However, it is necessary to explain to the patient that stopping smoking can lead to immediate benefits or even long-term benefits, as smoking cessation is undoubtedly linked to a reduced risk of cigarette smoking diseases and an overall improvement in health [50]. There are several pieces of scientific evidence to support this beneficial outcome, including a study of 34,000 British male doctors, which showed that the number of years of smoking affects health and mortality differently. Specifically, it has been observed that quitting smoking at the age of 50 years reduces the risk of smoking-related death two-fold, and that cessation at the age of 30 years avoids any risk. In addition, stopping smoking at the age of 60, 50, 40, or 30 years has led to gains of about three years, six years, nine years, and ten years of survival, respectively [70].

The impact of smoking cessation on fracture healing before treatment of a nonunion fracture was also assessed. Several studies have shown that the failure rate in the treatment of nonunion patients is higher in smokers [71,72]. Therefore, the orthopedic surgeon may choose not to perform the procedure until the patient stops smoking. Generally, a minimum of 24 h is recommended to eliminate CO from the blood and bring the carboxyhemoglobin back to normal values [50]. In this regard, Mills et al. have recently conducted a meta-analysis of randomized trials and observational studies to establish the effect of preoperative smoking cessation on postoperative complications and to determine whether there is an optimal cessation period before surgery [73]. The analysis found that smoking cessation reduces postoperative complications by about 40%, and that the longer the smoking cessation period before surgery, the greater the reduction in complications.

## 7. Discussion

Although cigarette smoking is the largest preventable cause of death in the world, the number of smokers is very high globally, suggesting that the clinical relevance of this important problem is still underestimated. Therefore, the aim of our review was to (i) summarize current knowledge on this topic and examine the impact of smoking on the skeleton, with particular reference to bone fractures; (ii) assess how the pathophysiological mechanisms responsible for the effect of smoking on bone tissue can impair the fracture healing process; and (iii) understand whether smoking cessation may have short and/or long-term effects on bone health.

Numerous clinical and experimental studies show that cigarette smoking has deleterious effects on the skeleton and worsens the prognosis of various orthopedic disorders and surgical procedures [11]. In particular, cigarette smoking has been shown to cause an imbalance in bone turnover mechanisms, leading to lower bone mass and lower BMD, both in young and elderly patients [27,74].

For hip fractures, it has been estimated that the increased risk caused by cigarette smoking has reached 40% in men and 31% in women [8,12]. In addition, mortality rates ranging from 20% to 24% were recorded in the year following a hip fracture, with an increased risk of death persisting up to five years later [75]. Considering these worrying data, smoking was identified as a risk factor for osteoporosis and fragility fractures, and was included in the Fracture Risk Assessment Tool [21].

The pathophysiological mechanisms by which smoking affects bone health are not yet well known, and the few studies aimed at clarifying the underlying cellular and molecular mechanisms are contradictory. Pathogenesis is quite complex and involves several processes, due to both direct and indirect effects of smoking on the activity of osteoblasts and osteoclasts. Indeed, it has been proposed that smoking can indirectly affect bone mass through the alteration of body weight, the PTH/vitamin D axis, adrenal hormones, sex hormones, and increased oxidative stress in bone tissue. Moreover, smoking can have a direct effect on bone mass, influencing the osteogenesis and angiogenesis of bone [21]. Among these, the RANKL-RANK-OPG pathway seems to play a key role in the processes by which smoking can alter bone health, as it is known to have a great influence on osteoclastogenesis and osteoclast function, as well as its interaction with most of the indirect pathophysiological mechanisms that influence bone turnover and bone mass [76,77].

Several studies have shown that there is a close and direct association between cigarette smoking, decreased BMD, and impaired fracture healing, even though the underlying mechanisms have not yet been fully understood. For example, it has been reported that smoke inhalation causes a decrease in the expression of angiogenic markers and compromises bone healing, as well as possibly leading to a delay in fracture union [58]. However, many substances contained in cigarettes have a negative effect on bone healing and it is not clear whether a single toxic substance or several toxic compounds may be responsible for these effects on bone tissue. In most cases, the negative effects of smoking on bones have been attributed to nicotine, the most pharmacologically active component in tobacco, which directly and indirectly affects cell metabolism [13].

In addition to altering the bone healing process, smoking also delays the healing time after fractures [52], and it is associated with several short-term postoperative complications responsible for longer hospital stays and increased consumption of resources [11]. Smoking cessation is therefore highly advisable to prevent the onset of bone metabolic diseases. In this regard, both short-term and long-term positive effects on bone metabolism have been found. In particular, the effects of smoking cessation in the short-term include an improvement in the fracture healing process, a reduction in the frequency of postoperative complications, and a reduction in hospitalization times. On the other hand, the positive long-term effects of smoking cessation include an increase in BMD at various bone sites and a reduced incidence of fragility fractures [78]. Although there are currently no precise time guidelines, researchers agree that the reduction in complications will be greater the longer the smoking cessation period before surgery. Therefore, encouraging patients to quit smoking several weeks before surgery is undoubtedly an important recommendation for patients.

## 8. Potential Intervention Strategies for the Prevention and Treatment of Smoking-Related Bone Damage

Although quitting smoking has undoubted health benefits in general and reduces the risk of smoking-related diseases, it is not yet clear whether quitting will reverse the effects on bone health. The health benefits of smoking cessation are obvious in the long term, but it is not clear whether the same is true for bone tissue. It is our opinion that quitting smoking, taking regular exercise, and starting physiotherapy can help to restore the function of the musculoskeletal system, even after many years of addiction. As mentioned above, several studies in the literature show that quitting smoking before surgery leads to a reduction in post-operative complications. It is our opinion that these beneficial effects occur mainly in spongy bone, which has a higher rate of bone turnover than cortical bone. Quitting smoking before surgery could trigger a mechanism in the spongy bone to improve bone quality and density. These beneficial effects certainly depend on the time between smoking cessation, the age of the patient, and the number of cigarettes smoked. Given this evidence, we believe that it is of considerable importance to carry out vigorous anti-smoking campaigns to inform the young population of the risks to the musculoskeletal system associated with cigarette smoking; since nicotine has a direct effect on osteoblasts, the cells that produce the bone matrix, young smokers are much more likely to develop a smaller skeletal system and to manifest painful and debilitating pathologies earlier than young non-smokers. In regard to medical therapy, treatment with vitamin D and anti-osteoporotic drugs, such as teriparatide and romosozumab, could help restore bone tissue quality by having an anabolic effect. In addition, it has been shown that hyperbaric chamber sessions may help to delay fracture consolidation: oxygen dissolves in the plasma, reaching every cell in the body in large quantities, a process that is undoubtedly compromised in a smoker, where the percentage of inhaled carbon monoxide increases and the percentage of oxygen decreases.

## 9. Conclusions

**Figure 2:**
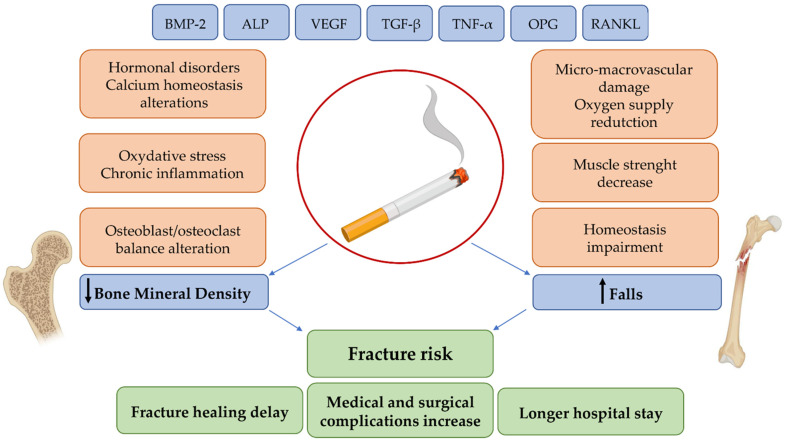
Schematic representation of the relationship between the biological effects and clinical consequences of smoking.

The effect of smoking on bone health is undoubtedly complex and variable, and involves various biological processes, which are largely unclear, with a considerable clinical and medical impact (Figure 2). Smoking is still one of the main causes of preventable death and disability and, despite the recent decline in prevalence, the number of smokers continues to increase due to population growth. As a result, the onset of smoking-related diseases may also increase, including an increased number of osteoporotic fractures. Therefore, as smoking continues to be a major public health concern, we believe that further research is needed in order to clarify the underlying mechanisms of action and to better understand this association between cigarette smoking and the onset of bone diseases. Finally, we suggest that smoking cessation can at least partially reverse the negative effects of smoking on the skeleton. Further research will also be needed to improve fracture risk prediction in smokers and to identify potential targets of pharmacological intervention.

## Figures and Tables

**Table 1:** Potential pathophysiological mechanisms by which smoking damages bone health.

**INDIRECT** **EFFECTS**	**Pathophysiological Mechanism**	**Methods**	**Bone Effects**	**References**
	Nicotine causes an alteration in body weight, suppressing appetite and increasing dopamine and serotonin levels	Height, weight, and waist circumference measurement Percent total body weight measurement via electrical bioimpedance	Weight loss causes a decrease in the mechanical load on the skeletal system, leading to a decrease in BMD	[23]
		Quantity of cigarettes smoked BMI measurement Waist circumference measurement		[24]
		Quantity of cigarettes smoked Appendicular skeletal muscle mass evaluation Percent of fat mass		[28]
		BMI measurement Body weight, waist and hip circumferences measurement Adipose cell metabolism and resting metabolic rate evaluation		[26]
	Tobacco suppresses PTH, cholecalciferol, and calcitriol production Nicotine increases skin aging, causing it to lose its capacity to synthesize cholecalciferol	25(OH)D levels measurement BMD at L2-L4 and proximal femur measurement Smoking habits, daily dietary calcium intake, and alcohol consumption evaluation	The reduction in serum levels of PTH and consequent vitamin D activation causes a decrease in BMD	[33]
		Gene expression analysis of *MMP-1* with qRT-PCR		[34]
		Smoking status evaluation Measurement of serum PTH levels Evaluation of intakes of calcium and vitamin D with a food-frequency questionnaire		[30]
	Smoking causes a decrease in testosterone levels Some alkaloids derived from tobacco inhibit the activity of the aromatic enzyme, exerting an antiestrogenic effects	Steroid hormone levels measurement	The decrease in gonadal hormone levels causes a reduction in the activity and proliferation of osteoblasts and an increase in the resorption activity of osteoclasts	[35]
		Radiometric assay of estradiol 2-hydroxlation		[36]
	Smoking causes an increase in levels of adrenocorticotrophin, causing a consequent increase in circulating cortisol levels	Evaluation of the number of cigarettes smoked Saliva sample collection for the assessment of cortisol	Cortisol causes a reduction in collagen and bone matrix synthesis	[41]
**DIRECT** **EFFECTS**	Smoking causes an increase in the RANKL/OPG ratio	Smoking status and physical activity assessment Blood specimen collection to determine serum levels of bone turnover markers	Increase in osteoclastogenesis and bone resorption	[45]
		Gene expression analysis of *RANK/RANKL/OPG* with qRT-PCR in rat model		[46]
	Smoking causes an increase in DKK1 (bone formation inhibitor) levels and a decrease in P1NP (bone formation marker) levels	BMD measurement Serum analyses of CTX-1, P1NP, OPG, RANKL, DKK1, Sclerostin, TNF-α, and Leptin	Alteration of bone formation	[30]

BMI: bone mass index; BMD: bone mineral density; PTH: parathyroid hormone; MMP-1: metalloproteinase 1; qRT-PCR: quantitative real-time polymerase chain reaction; RANK: receptor activator of nuclear factor kappa-B; RANKL: receptor activator of nuclear factor kappa-B ligand; OPG: osteoprotegerin; DKK1: dickkopf-1; CTX-1: C-terminal telopeptide of collagen type 1; P1NP: N-terminal propeptide of procollagen; TNF-α: tumor necrosis factor-alpha.

## Data Availability

No new data were created or analyzed in this study. Data sharing is not applicable to this article.
